# A case report of prostatic malacoplakia after heart transplantation and review of the literature

**DOI:** 10.1093/jscr/rjaf716

**Published:** 2025-09-11

**Authors:** Shangzhen Geng, Lihua Li, Yunji Sun, Jianchao Zhang, Yunwei Li, Li Chen

**Affiliations:** Department of Urology, Shandong Provincial Third Hospital, No. 12, Wuyingshan Middle Road, Tianqiao District, Jinan, Shandong Province 250031, China; Department of Urology, Shandong Provincial Third Hospital, No. 12, Wuyingshan Middle Road, Tianqiao District, Jinan, Shandong Province 250031, China; Department of Urology, Shandong Provincial Third Hospital, No. 12, Wuyingshan Middle Road, Tianqiao District, Jinan, Shandong Province 250031, China; Department of Urology, Shandong Provincial Third Hospital, No. 12, Wuyingshan Middle Road, Tianqiao District, Jinan, Shandong Province 250031, China; Department of Urology, Shandong Provincial Third Hospital, No. 12, Wuyingshan Middle Road, Tianqiao District, Jinan, Shandong Province 250031, China; Public Health Department, Shandong Provincial Third Hospital, No. 12, Wuyingshan Middle Road, Tianqiao District, Jinan, Shandong Province 250031, China

**Keywords:** prostatic malacoplakia, post-heart transplantation, three-groove and three-arc method, prostate laser enucleation, thulium laser

## Abstract

Prostatic malacoplakia is a relatively rare granulomatous inflammatory disease that is easily misdiagnosed as prostate cancer. Usually, patients’ symptoms are improved through antibacterial treatment. We performed thulium laser enucleation of the prostate by the triple-groove, triple-arc method on a 68-year-old man, a patient with significant dysuria. The patient has significantly improved lower urinary tract symptoms and quality of life.

## Introduction

Malacoplakia is a rare granulomatous inflammatory lesion first reported by Michaelis and Gutmann in 1902. It commonly occurs in the urinary, reproductive, and digestive systems, with the bladder being the most frequently affected site [1]. This study reports a case of prostatic malacoplakia in a patient after heart transplantation. The patient underwent three-groove and three-arc prostate laser enucleation, which resolved his symptoms of dysuria and nocturia. The patient recovered well postoperatively.

## Case report

**Figure 1:**
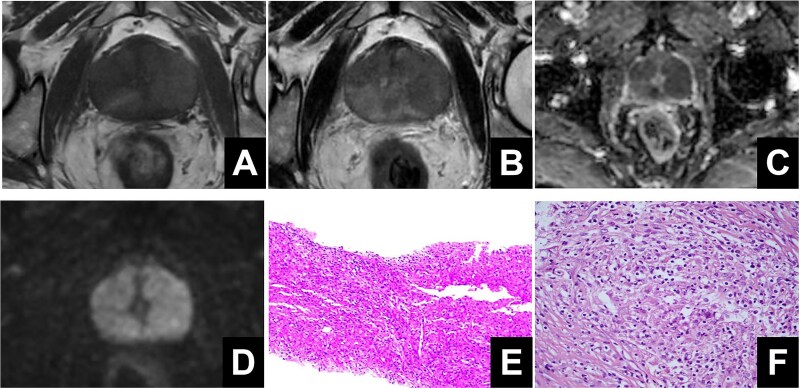
(A) High signal on *T*_1_-weighted imaging. (B) Low signals on *T*_2_-weighted imaging. (C, D) Restricted diffusion was observed on diffusion-weighted imaging and apparent diffusion coefficient. (E) The pathological results of transperineal prostate puncture surgery. (F) The pathological results of prostate laser enucleation surgery.

An elderly male patient was admitted due to “difficulty urinating and increased nocturia for 3 years.” Digital rectal examination revealed symmetric enlargement of both lateral lobes of the prostate, disappearance of the central sulcus, firm texture, no tenderness, and no palpable nodules. Prostate-specific antigen (PSA) was 1.810 ng/ml; urinary flow rate was 5.9 ml/s, urinary system ultrasound showed a prostate size of ~4.5 × 4.0 × 3.6 cm, and prostate MRI indicated unclear boundaries between the central gland and peripheral zone, with multiple patchy low signals on *T*_2_-weighted imaging (T2WI) and fat-suppressed imaging, and high signals on diffusion-weighted imaging (DWI) with reduced apparent diffusion coefficient (ADC) values, suggesting prostate cancer (Fig. 1A–D). The Prostate Imaging Reporting and Data System (PI-RADS) score was 5. A transperineal prostate biopsy under ultrasound guidance was performed, and pathological examination revealed a large number of epithelioid cells with eosinophilic cytoplasm, accompanied by lymphocytes, plasma cells, and neutrophils. Immunohistochemistry showed S100 (indicating the presence of Michaelis–Gutmann bodies), CD10, and CD68, consistent with malacoplakia (Fig. 1E). The patient’s urine white blood cell count was 419.1/ul. After antibacterial treatment, the infection was cured, but the patient still experienced difficulty urinating. To improve the clinical symptoms, three-groove and three-arc prostate thulium laser enucleation (Raykeen, China) was performed. Postoperatively, the patient’s urination was smooth, with a urinary flow rate of 18.2 ml/s. Postoperative pathology confirmed prostatic malacoplakia, with special staining showing PAS (Michaelis–Gutmann bodies+) (Fig. 1F). The work has been reported in line with the Surgical CAse REport (SCARE) criteria [2].

## Discussion

Malacoplakia was first discovered in the bladder by Michaelis and Gutmann, and later reported in the prostate by Carruthers *et al.* [3]. It is a rare chronic inflammatory disease, more common in immunocompromised individuals, with a female-to-male incidence ratio of 4:1. The disease most frequently affects the genitourinary system, particularly the bladder, but can also occur in other systems such as the colorectum, lungs, and thyroid. The hallmark histological feature is the presence of basophilic Michaelis–Gutmann bodies, which result from incomplete bacterial degradation due to abnormal macrophage function [3].

The pathogenesis of malacoplakia remains unclear, with three main hypotheses: (i) urinary tract infection, primarily caused by *Escherichia coli* or other Gram-negative bacteria; (ii) immune suppression, as malacoplakia often occurs in immunocompromised patients, such as those with HIV or organ transplants; (iii) abnormal lysosomal function in macrophages, leading to impaired bacterial digestion and the deposition of iron and calcium, resulting in the formation of Michaelis–Gutmann bodies [4, 5]. This patient had a 22-year history of diabetes and underwent heart transplantation 9 years ago due to heart failure, followed by immunosuppressive therapy. However, urine culture results were negative, and no *E. coli* infection was detected.

The clinical manifestations of malacoplakia vary depending on the affected site. When it occurs in the prostate, symptoms may include urinary frequency, urgency, dysuria, urinary retention, fever, and chills. Prostatic malakoplakia is similar to prostate cancer in that it can cause an elevation in PSA levels and may show imaging changes in the peripheral zone of the prostate [6, 7]. MRI findings often show high signals on *T*_1_-weighted imaging, low to intermediate signals on T2WI, high signals on DWI, and low ADC values, with a PI-RADS score of 4–5 [8].

Clinically, prostate laser enucleation is a standard surgical treatment for benign prostatic hyperplasia, involving the removal of hyperplastic tissue along the prostate capsule to relieve symptoms. It is the removal of hyperplastic prostate tissue along the surgical envelope of the prostate. For malacoplakia, antibacterial treatment, such as fluoroquinolone antibiotics [9], may be administered, and surgery may be necessary in some cases [10]. In this patient, the elevated urine white blood cell count was treated with levofloxacin, and the urine routine returned to normal. However, due to persistent dysuria and urinary retention, three-groove and three-arc transurethral prostate laser enucleation was performed. This modified three-lobe prostate enucleation technique involves three grooves at the 5, 7, and 12 o’clock positions connecting the bladder neck and verumontanum, dividing the prostate into left, right, and middle lobes. The three arcs connect the 5–7, 7–12, and 12–5 o’clock positions at the verumontanum plane, allowing enucleation along the surgical capsule. The urinary catheter was removed on the third postoperative day, and the patient’s urinary symptoms significantly improved, with a maximum urinary flow rate of 18.2 ml/s.

The three-arc method of prostate laser enucleation is a modified version of the traditional trilobar laser enucleation of the prostate. This procedure makes it easier to find the prostate surgical envelope by first looking for it in front of the seminal mound. The three grooves and three arcs can initially dissociate the mucosa and can adequately prepare the prostate tissue for the next step of enucleation. Therefore, the procedure reduces the learning curve for beginners. There are few reports of thulium laser enucleation of the prostate by the triple-groove, triple-arc method for prostatic malacoplakia. We found that this surgical procedure has the advantages of high tissue vaporization and cutting ability, low intraoperative bleeding, and a short learning curve, making it a safe and effective treatment modality.

## Conclusion

In conclusion, prostatic malacoplakia is a rare chronic inflammatory disease that can present with lower urinary tract symptoms, elevated PSA, urinary tract infection, and imaging abnormalities. Antibacterial treatment is recommended, and for patients with significant dysuria, thulium laser enucleation of the prostate by the triple-groove, triple-arc method can be performed after antibacterial therapy to significantly improve lower urinary tract symptoms and quality of life.
